# Minimally Invasive Subtemporal Intradural Approach for Penetrating Orbitocranial Injury by Wooden Foreign Body Into the Lateral Wall of the Cavernous Sinus

**DOI:** 10.3389/fsurg.2020.533567

**Published:** 2020-09-22

**Authors:** Elad Avraham, Alexander Smolikov, Rozalia Smolyakov, Amit Azriel, Yuval Sufaro, Tehila Kaisman-Elbaz, Gregory Zlatin, Israel Melamed

**Affiliations:** ^1^Department of Neurosurgery, Soroka University Medical Center, Beersheba, Israel; ^2^Department of Radiology, Soroka University Medical Center, Beersheba, Israel; ^3^Infectious Diseases Unit, Soroka University Medical Center, Beersheba, Israel; ^4^Department of Otorhinolaryngology (ENT), Soroka University Medical Center, Beersheba, Israel

**Keywords:** orbitocranial injury, cavernous sinus, penetrating injury, minimally invasive, subtemporal approach, wooden foreign body

## Abstract

Non-missile transorbital penetrating head injuries are relatively rare, though potentially fatal injuries. Trajectory for intracranial entrance is typically via the orbital roof, the superior orbital fissure (SOF), or the optic canal. Non-metallic intracranial penetrating injuries are even scarcer and may pose unusual diagnostic and surgical challenges. Here we present and discuss a unique case of a penetrating injury by a wooden foreign body (FB) which entered and expanded the inter-dural space of the lateral cavernous sinus (CS) sinus wall without intracavernous or intradural involvement. The patient was a 71 year-old male who fell face-down and sustained a penetrating transorbital injury by a dry twig fragment, which passed through the SOF and into the interdural space of lateral wall of the ipsilateral CS. The patient was fully conscious (GCS15) at presentation but had severe ocular injury (complete ophthalmoplegia and blindness of the injured eye). The wooden FB was successfully removed via a minimally invasive subtemporal intradural approach with no apparent immediate or long-term complications. We emphasize the unusual diagnostic and surgical challenges related to this kind of rare injuries as reflected by the decision-making considerations taken in the presented case.

## Introduction

Transorbital intracranial injuries are rare and potentially fatal (1, 2). This type of injury, represents 0.04% of all head trauma, 24% of all penetrating head trauma in adults, and 45% in the pediatric age group (3). Penetrating orbital injury may lead to severe brain injury when the FB enters the cranium and is associated with high rates of orbital and cerebral complications (4–11). Due to the fact that the orbit is a pyramid shaped structure, the penetrating FB is usually directed toward the apex of the orbit and thus can access the intracranial space, trough the optic nerve foramen or SOF, without damaging the surrounded orbital bones (3). Diagnosis can be challenging in cases of trivial trauma. The radiological diagnosis can be particularly challenging for a wooden FB which can mimic pneumocephalus in CT scans (12–17).

Here we present and discuss a unique case of a penetrating wooden FB injury located within the interdural space of the lateral CS wall without intracavernous or intradural involvement.

## Case Report

### Presentation

Under the influence of alcohol intoxication, a 71 year-old male fell face-down at the edge of an agricultural field. After returning home, his children have noticed a prominent swelling around his left eye. He refused any medical attention for 2 days. finally, 3 days post-injury, he presented to the emergency department with fever and severe left periorbital edema accompanied with a purulent discharge. The patient was fully conscious, though uncooperative. A small superior eyelid puncture wound around the left medial cantus was observed. Neurological examination revealed complete left ophthalmoplegia and blindness (no light perception) of the left eye. Fundoscopy revealed a pale left optic nerve. No signs of meningeal irritation were observed. Sensory functions of the left trigeminal nerve were hard to evaluate (due to poor cooperation by the patient). Muscles of mastication functions were preserved.

**Figure 1:**
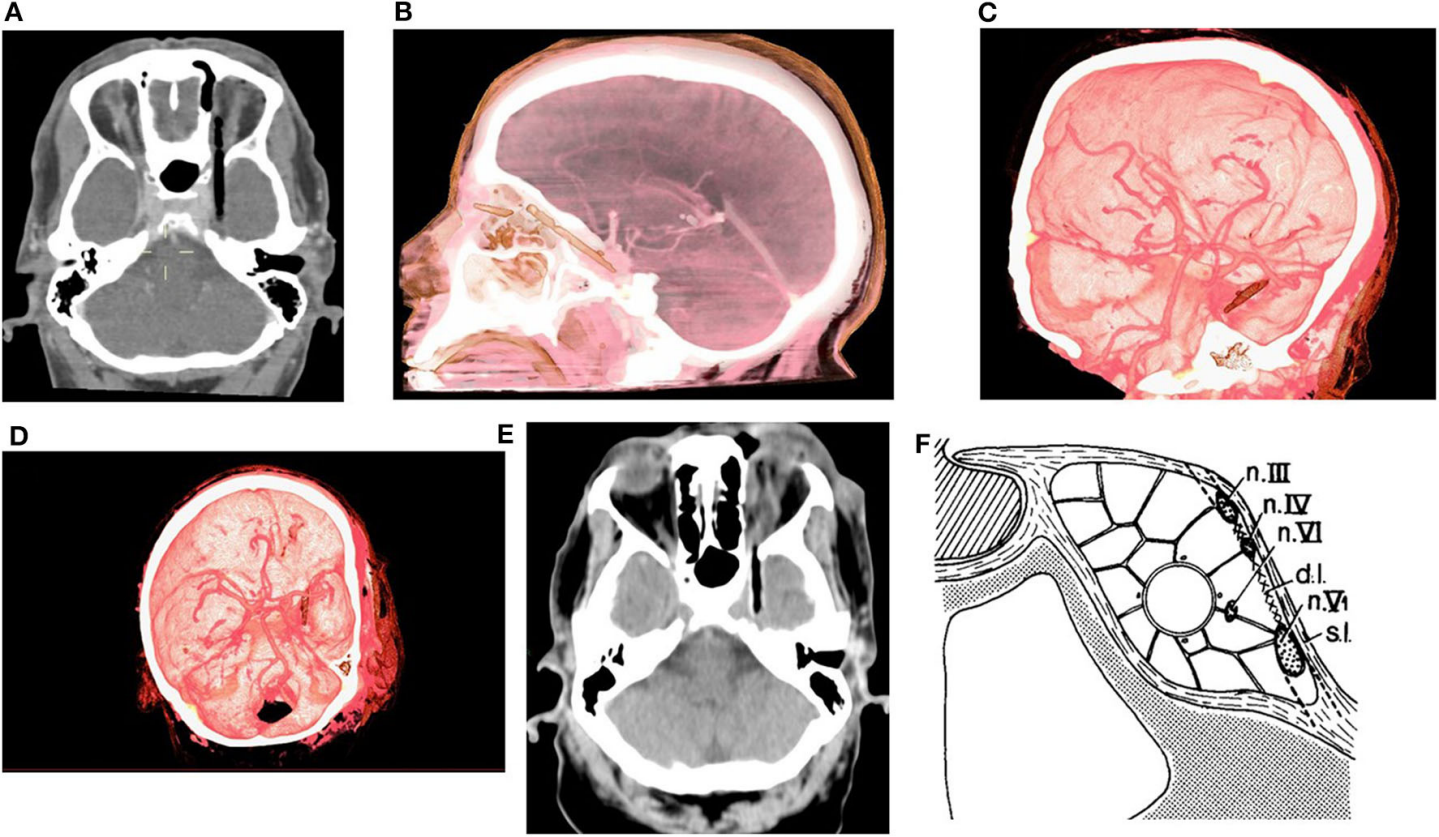
**(A)** Pre-op CT scan axial view, **(B)** Pre-op CTA 3D reconstruction lateral view, **(C,D)** Pre-op CTA 3D reconstruction oblique views. **(E)** Axial view from CT scan which was performed after the orbital stage of the surgery. **(F)** Lateral wall of CS [s.l, superficial layer; d.l, deep layer; Modified from (18), with permission].

Non-contrast CT of the head was performed and revealed a linear shaped, segmented into 2 pieces (short superficial part and longer deep part) hypodense, air-mimicking FB, extending from the upper medial part of the left orbit (Zone 3b according to classification of Turbin R. and al), through the SOF (sparing the globe) and into the cranial space (Figures 1A,B). Prompt discussion with a senior neuro-radiologist was performed and the following surgical related aspects were concluded:

- No evidence of facial or cranial bones fractures.
- The FB is located lateral to the left carvernous sinus with no evidence of intra cavernous involvement. Extra-cavernous-Intra-dural location of the FB was assumed.
- No evidence of active bleeding, vascular injury, or CS thrombosis in the CTA and CTV studies (Figures 1C,D).

The patient was planned for a 2-staged surgery—first, maximal safe removal of the orbital part of the FB (by the ENT team), followed by a second intra-cranial procedure.

### The Surgery – Orbital Stage

The ENT surgeon opened and expanded the small palpebral entry wound and performed intra-orbital exploration. The superficial part and only the proximal end of the deep part of the FB (which was found out to be a dry twig) could be resected via this approach. Pus evacuation and meticulous intraorbital irrigation were performed. The impression of the ENT surgeon at that stage was that the residual intraorbital FB is stuck and further removal of it is highly dangerous. Therefore, head CT scan was performed (Figure 1E) the patient was transferred to the neurosurgical operating theater for the intracranial stage.

### The Surgery – Intracranial Stage

**Figure 2:**
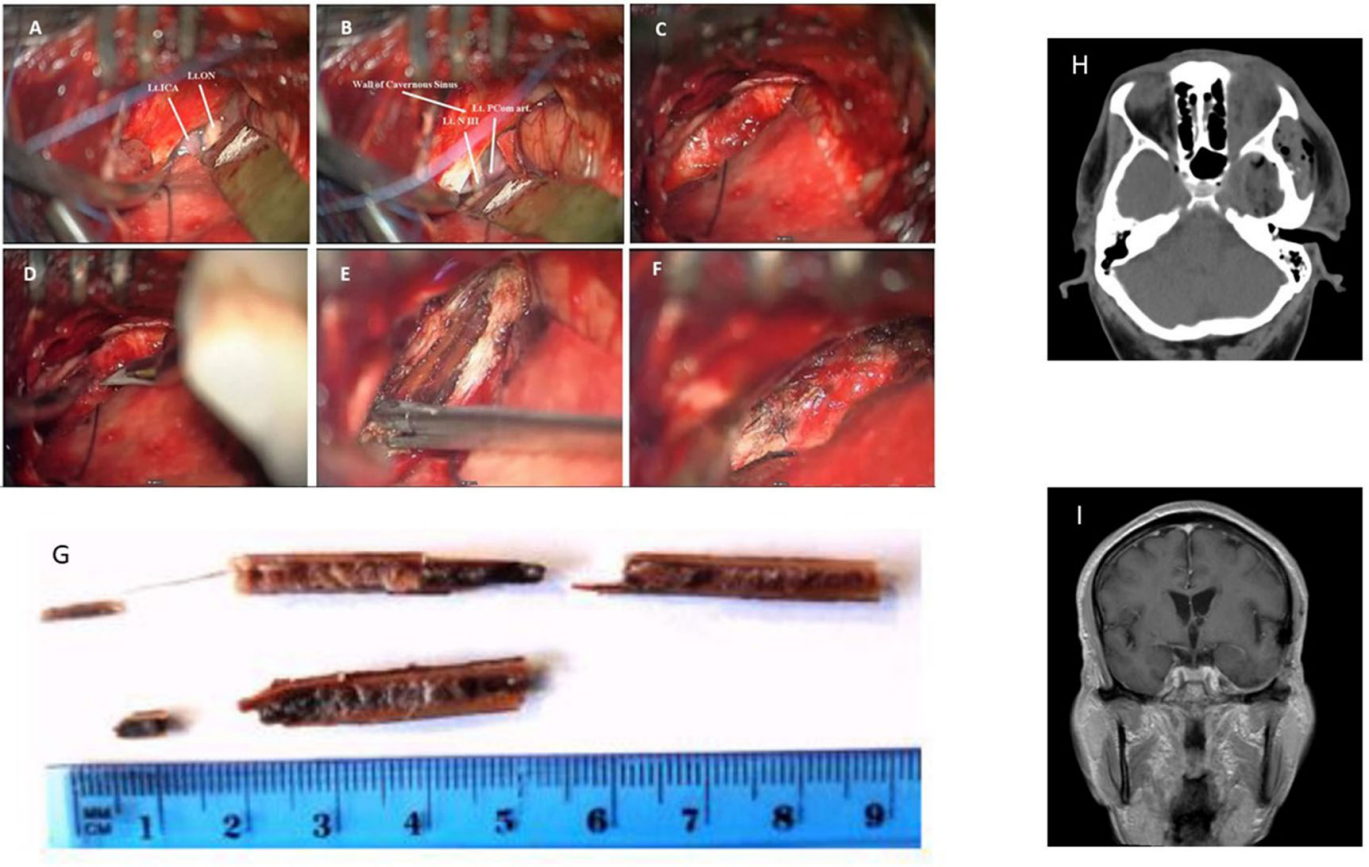
Intra-operative images: **(A,B)** Left CS lateral wall and adjacent non-injured anatomical structures: Lt. ICA—left Internal Carotid Artery, Lt. ON—left Optic Nerve, Lt. PCom—left Posterior Communicating Artery, Lt. NIII—left Oculomotor Nerve. **(C)** Superficial layer of CS lateral wall. **(D)** Opening of superficial layer of lateral wall of CS, and **(E)** Identification and removal of the FB. **(F)** Sutured superficial layer of CS lateral wall. **(G)** Photograph of the removed wooden FB fragments. **(H)** Post-op CT **(I)** Follow-up MRI scan a year after the injury.

The patient was placed in the supine position with his head fixated by a Mayfield head clamp and rotated to the right. Small subtemporal craniotomy (2.5 × 2.5 cm) through a small linear scalp incision was performed. To minimize subsequent temporal lobe retraction, CSF drainage trough a small dural incision was performed at this early stage, followed by drilling of the temporobasal edge of the craniotomy window with high speed drill. The dura was incised in a “C-shaped” fashion. Gentle retraction of the temporal lobe and additional CSF drainage allowed for the exposure of the thickened hyperemic superficial layer of the lateral wall of the CS. In contrast to our expectations the FB was not visualized at this stage. Adjacent non-injured anatomical structures were recognized, namely, the mesial part of the temporal lobe, the tentorial edge, the left optic nerve, the left ICA and PCom, and the left oculomotor nerve (Figures 2A,B). Careful inspection of CS lateral wall under high magnification allowed visualizing the prominence related to the FB lying beyond the superficial layer of the lateral wall of the CS (Figure 2C). This layer was opened by a linear incision along this prominence (Figure 2D), thereby exposing the FB—a split dry twig (Figure 2E). As opposed to the explicit pus that was seen and evacuated from the orbit, no accompanied pus discharges were found around the intracranial (interdural) part of the FB. With the aid of micro-pituitary forceps, fragments were removed in a staged manner. The movement of the forceps was strictly directed along the axis of the FB. The residual orbital part of the fragment was pulled out posteriorly and was removed with no noticeable resistance. The total length of the fragments that were removed was in accordance with the pre-operative estimated size of the FB (Figure 2G). Further exploration of the surgical field did not reveal any residual fragments, and complete resection was assumed. The intact deep layer of the lateral CS wall was exposed. A minor oozing was observed after the last piece of the FB was removed, which was controlled by the application of a small piece of SURGICEL®. After meticulous irrigation, the incision in the superficial layer was closed in a watertight fashion (continuous 7/0 Prolene suture, Figure 2F). The dural incision was also closed in a watertight fashion (continuous 5/0 prolene suture). Bone flap was placed back and fixed with two titanium CranioFix clamps (CranioFix®2).

### Postoperative Course

Post-operative course was uneventful. Post-operative CT scan demonstrated complete removal of the FB (Figure 2H). The periorbital edema and purulent ocular discharges resolved shortly after the surgery. No improvement in the complete left ophthalmoplegia and blindness were observed. Broad spectrum empiric IV antibacterial therapy that was started before the surgery was continued, followed by a targeted therapy with Ceftriaxone 2 g × 2/day for an additional 10 days (as was instructed by our institutional infectious disease unit after the results of ceftriaxone-sensitive *Enterobacter* species growth in the microbiological assay of the pus sampled during intraorbital surgery). The patient was discharged home about 2 weeks after presentation. During the follow-up period no infectious-related complications (ocular or CNS) were observed. The patient did not have any suspected seizure events nor any apparent cognitive deterioration. Follow-up MRI scan a year after the injury did not demonstrate any pathological findings other than mild gliosis detected at the basal temporal lobe (Figure 2I). There were no clinical nor radiological evidence of late vascular sequelae of the insult (such as carotid-cavernous fistula or dural AVF). Complete ophthalmoplegia and blindness of the left eye persisted at 1 year follow-up. Painless hypoesthesia at the left V1 and V2 territories was observed (those symptoms were probably present but hard to deduce at presentation). No other neurological deficits were observed.

## Discussion

Orbito-cranial penetrating injuries are relatively rare. The vast majority of patients suffering such injuries are within the pediatric age group and falling is the main mechanism (1). Injuries involving a wooden FB are even scarcer than those involving other materials such as metal or glass and may pose unusual diagnostic and surgical challenges (12–17, 19). Table 1 highlights a list of selected published cases of wooden FB orbito-cranial penetrating injury. Here we presented a rare case in which the wooden FB entered and expanded the inter-dural space of the lateral CS sinus wall without intracavernous or intradural involvement.

**Table 1:** Selected published cases of orbito-cranial penetrating injury by a wooden FB.

**Author**	**Age (years) and gender**	**Injury description**	**Approach and result**
Matsuyama et al. (10)	1, M	Chopstick; via orbital apex approaching pre pontine cistern	Early surgery (12 h); frontotemporal craniotomy with orbital and optic canal unroofing for removal of fragments and purulent discharge evacuation + ABX; good recovery
Maruya et al. (8)	56, F	Bamboo fragments; via lateral orbital wall to ipsilateral temporal wall	Delayed (2 weeks) diagnosis and surgical intervention resulted with brain abscess; frontotemporal craniotomy and orbitozygomatic osteotomy for removal of foreign bodies + stereotactic aspiration of brain abscess and long-term ABX; good recovery
Dunn et al. (6)	53, F	3.0 cm × 0.5 cm wooden fragment intra orbital to orbital apex	Early Intra orbital exploration and removal of the fragment; post-operative ABX treatment; good recovery
Al-Otaibi and Baeesa (19)	4, F	Pencil via medial orbital roof to inferior frontal lobe	Delayed presentation and diagnosis; frontal brain abscess and subdural empyema; supraorbital minicraniotomy for removal of foreign body and abscess and empyema evacuation + duraplasty; long-term ABX; good recovery
Parajuli et al. (20)	14, F	Large bamboo fragment; through lower eyelid and orbital floor via orbital apex and cavernous sinus with compression of the paraclinoid intradural portion of the left internal carotid artery	Early Frontotemporal craniotomy for fragments removal and dural repair + intra orbital exploration; ABX; no light perception and nearly complete ophthalmoplegia in the affected eye (as described at presentation). Otherwise, good recovery

Due to dry wood's air-filled porous microstructure, it mimics pneumocephalus on CT scans (both in soft and bone windows), therefore raising a radiological diagnostic challenge for intracranial wood injuries (17, 21). Helpful differentiating features of wooden FB from pneumocephalus is the typical linear appearance of the FB, and different attenuation values–dry wood measures typically in the −100 to −170 HU range while pneumocephalus measures in the −600 to −1000 range (17). To note, CT attenuation values vary with the water content of the wood, and so a freshly cut wood which has a relatively high water content may mimic soft tissues in CT images (21). Since surgery introduce air into the area of interest the benefit of intra-operative CT scan for evaluation of residual fragments is limited. Therefore, a thorough pre-operative evaluation of the shape and size of the fragments is warranted. MRI is a safe modality (as long as no metallic FB involvement is suspected) with higher sensitivity (compared to CT) for detecting wood FB (22).

Wood, with its porous consistency and organic nature, serves as a medium for microbial proliferation, and thus may increase the risks to complications such as panophthalmitis, abscess, and Fistula (23) (compared to other FB materials), thus highlighting the importance of early and complete resection of the wooden FB, with the additional benefit of pathogen identification for an early tailored antibiotic treatment.

In their pivotal article (18), Umansky and Hilel demonstrated beautifully the anatomy of the lateral CS wall, which was not fully clear at that point, and showed it to be formed of two separated layers—a superficial dural layer (dura propria/meningeal dural layer which is continuous with middle fossa meningeal dural layer) and a deep layer formed by the sheaths of nerves III, IV, and V1 (and to variable extent V2) and a reticular membrane extending between the sheaths. Hence, Cranial nerves III, IV, V1 (±V2) are embedded within the deep layer, while cranial nerve VI and the intra-cavernous part of the ICA (C4) travel through the CS (18, 24–26) (Figure 1F). At the SOF (as well as in any other foramen\fissure where cranial nerves\vessels exit the cranial vault) the periosteal dura folds back along the bone and continues as the extracranial periosteum, whereas the dura propria remains intracranial (27). Therefore, a space (filled with loose connective tissue) is formed intracranially between the dura propria and the epineurium of the cranial nerves. At the SOF this space is continuous and relatively prominent forming the inter-dural space of the CS lateral wall. Extracranially it is continuous with the space between the extracranial\orbital periosteum and epineurium of the cranial nerves (28). The meningo-orbital band (MOB), which is basically the fusion of the SOF lateral upper border periosteum with the lateral inferior border periosteum, forms a partial separation between those spaces. In the current case, it seems that the FB most likely entered the inter-dural space of CS lateral wall through the SOF and the MOB expanding this above-mentioned space whilst preserving both the deep and superficial layers of the CS, thus sparing both intracavernous and intradural spaces.

Modern brain CT scan reconstructions allow for the acquisition of high-resolution imaging of the anatomical structures and their spatial relationship with the FB. In the current case, CT/CTA/CTV reconstructions allowed for realization that the FB trajectory spared the CS. Nevertheless, we falsely concluded that the FB penetrated the dura propria and thus has intradural extension. Retrospectively, careful reinspection of the pre-op CT allows to deduce the integrity of the dura propria and thus the accurate location of the FB. A priori correct deduction of the FB inter-dural location would have made the subtemporal extra-dural approach a reasonable alternative (as discussed below). Careful inspection of the pre-operative CTA can yield important insights regarding the spatial relationship between the FB and the nearby arterial structures. For marginal cases, in which higher resolution demonstration of the vessels is required, the utilization of formal angiography and\or MRA\MRV is warranted. If vascular damage is suspected, the surgical strategy should consider early proximal and distal control of the main vessels, or alternatively, pre-surgical endovascular intervention with conventional angiogram for intravascular inflatable balloon insertion (29). In the current case, the pre-operative assessment of the CTA scan indicated no involvement/injury of the left ICA or its branches with sufficient distance from the intradural segments of the ICA, and with preserved CS lateral wall (found in the surgery to be the deep layer) which separated FB from the intracavernous ICA. Therefore, the risk for intra-operative ICA injury was evaluated pre-operatively to be low, and we did not consider surgical\endovascular proximal ICA securing as mandatory.

The medial aspect of the temporal lobe and the lateral CS can be approached from different directions\craniotomies (e.g., fronto-temporal, supra-orbital, subtemporal) (18, 24, 25, 30). The subtemporal approach that we have chosen for the current case is ideal for a panoramic exposure of the CS as to allow sufficient visualization, control and safe microsurgical manipulations during all stages of surgery: evaluation and identification of the location of the FB located between the layers of the CS, opening the dura propria layer of the lateral wall of CS, complete removal of the FB (with spacious enough exposure as to apply posteriorly directed pulling movements) and finally, meticulous suturing of the dura propria layer of the sinus. The relatively long working distance to the CS makes it ideal for the utilization of the minimally invasive keyhole concept developed and perfected by A. Perneczky (31) in order to minimize the surgically induced trauma. Therefore, a small craniotomy located closest as possible to the temporal base (accompanied with drilling of the temporobasal edge of the craniotomy for minimization of temporal lobe retraction) was more than sufficient.

As mentioned above, if we were to know the correct location of the FB in the inter-dural space of the lateral wall of the CS pre-operatively, we would have consider utilizing an extradural approach as a reasonable alternative to the intradural approach that we chose. We consider the lack of direct intradural basal temporal lobe manipulation, which may result in the development of undesirable neurological sequalae [e.g., epilepsy, neurological deficits such as visual agnosia and prosopagnosia (32, 33)] as the principle advantage of the extradural approach. Nevertheless, we believe that for the current case, the intradural approach was the better choice, mainly due to the following reasons:

- It provides the mandatory spacious exposure required for the safe visualization and removal of the FB.
- It allows for a more perpendicular working angle with regard to the axis of the FB which in turn allows for pulling movements along this axis.
- It is less complex and less destructive [e.g., no need for MOB detachment and anterior clinoidectomy which are mandatory stages for sufficient dural retraction for the extradural approach (28, 30, 34)].

The main pros and cons considerations for the utilization of each approach for the current case are summarized in the Table 2.

**Table 2:** Main considerations for choosing between intradural and extradural approaches for resection of FB located along the interdural space of the lateral CS wall.

**Intradural approach**		**Extradural approach**	
**Advantages**	**Disadvantages**	**Advantages**	**Disadvantages**
Panoramic view of the CS	Temporal lobe retraction during manipulations	No direct intradural manipulation on basal temporal lobe	Suboptimal exposure
Perpendicular working angle	Risk of post-surgery CSF leak (though very low)	No Risk of post-surgery CSF leak	Suboptimal working angles
Less complex	No proximal control to ICA	Proximal control to ICA	Complex, including extradural drilling of sphenoid and temporal bones and anterior clinoidectomy.
Less destructive			Risk for injury of ICA, oculomotor and optic nerve during skull base drilling

We believe that complete resolution of the infection by a relatively short course of targeted IV antibiotic treatment can be attributed mainly to the following factors:

- The infection seemed to be restricted to the extracranial\orbital space.
- The unusual location of the FB between the two layers sparing both the CS and intradural space.
- Specifically, it is likely to assume that the preserved superficial layer lowered dramatically the risk of local spreading of the infection into the adjacent CS sinus septic thrombophlebitis
- Meticulous orbital and intracranial irrigation.
- Watertight suturing of the dura propria at the end of the surgery.
- Targeted antibiotic treatment post-operatively.

In conclusion, transorbital penetrating cranial injury by a wooden FB is rare and poses the risk for potentially life-threatening complications. Careful anamnesis, physical examination and inspection of pre-operative imaging accompanied by multidisciplinary approach (neurosurgery, neuroradiology, otorhinolaryngology, ophthalmology) are of highly importance for optimal emergent surgical planning and execution leading to good outcome for the patients.

## Data Availability Statement

The raw data supporting the conclusions of this article will be made available by the authors, without undue reservation.

## Ethics Statement

The Institutional Review Board of Soroka Medical Center waived the requirement for informed consent for the publication of this case report.

## Author Contributions

EA, AS, and IM: study conception, data collection, data analysis, manuscript writing/editing, and final approval of manuscript. RS, AA, YS, TK-E, and GZ: data collection, data analysis, manuscript editing, and final approval of manuscript. All authors contributed to the article and approved the submitted version.

## Conflict of Interest

The authors declare that the research was conducted in the absence of any commercial or financial relationships that could be construed as a potential conflict of interest.

## References

[1] Arslan M Eseoglu M Güdü BO Demir I. Transorbital orbitocranial penetrating injury caused by a metal bar. J Neurosci Rural Pract. (2012) 3:178–81. 10.4103/0976-3147.98228 22865972 PMC3409991

[2] Lin HL Lee HC Cho DY. Management of transorbital brain injury. J Chin Med Assoc. (2007) 70:36–8. 10.1016/S1726-4901(09)70299-0 17276932

[3] Kitakami A Kirikae M Kuroda K Ogawa A. Transorbital-transpetrosal penetrating cerebellar injury: case report. Neurol Med Chir. (1999) 39:150–2. 10.2176/nmc.39.150 10193148

[4] Arifin M Gill A Faried A. Penetrating skull fracture by a wooden object: management dilemmas and literature review. Asian J Neurosurg. (2012) 7:131–4. 10.4103/1793-5482.103716 23293668 PMC3532759

[5] Ahmed S Dutta D Civilian nonmissile penetrating brain injury. Apollo Med. (2018) 15:152–7. 10.4103/am.am_61_18

[6] Dunn I Kim D Rubin P Blinder R Gates J Golby A. Orbitocranial wooden foreign body: a pre-, Intra-, and postoperative chronicle: case report. Neurosurgery. (2009) 65:383–4. 10.1227/01.NEU.0000347474.69080.A1 19625895

[7] Lee JE Lee HY. A case of retained wooden foreign body in orbit. Korean J Ophtalmol. (2002) 16:114–8. 10.3341/kjo.2002.16.2.114 12546450

[8] Maruya J Yamamoto Y Wakay M Kaneko U. Brain abscess following transorbital penetrating injury due to bamboo fragments. Case report. Neurol Med Chir. (2002) 42:142–6. 10.2176/nmc.42.143 11936059

[9] Matsumoto S Hasuo K Mizushima A Mihara F Fukui M Shirouzu T. Intracranial penetrating injuries via the optic canal. Am J Neuroradiol. (1998) 19:1163–5. 9672032 PMC8338635

[10] Matsuyama T Okushi K Nogami K Hata M Murao Y. Transorbital penetrating intracranial injury by a chopstick: case report. Neurol Med Chir. (2001) 41:345–8. 10.2176/nmc.41.345 11487998

[11] Mutlukan E Fleck B Cullen J Whittle I. Case of penetrating orbitocranial injury caused by wood. Br J Ophthalmol. (1991) 75:374–6. 10.1136/bjo.75.6.374 2043585 PMC1042388

[12] Greaney M. Bamboo orbital foreign body mimicking air on computed tomography. Eye. (1994) 8:713–4. 10.1038/eye.1994.185 7867845

[13] Hansen J Gudeman S Holgate R Saunders R. Penetrating intracranial wood wounds: clinical limitations of computerized tomography. J Neurosurg. (1988) 68:752–6. 10.3171/jns.1988.68.5.0752 3357035

[14] Kahler R Tomlinson F Eisen D Masel J. Orbitocranial penetration by a fern: case report. Neurosurgery. (1998) 42:1370–3. 10.1097/00006123-199806000-00108 9632198

[15] Kim DH Park ES Seong HY Park JB Kwon SC Sim HB. A case of intracranial wooden foreign body: mimicking pneumocephalus. Korean J Neurotrauma. (2016) 12:144–7. 10.13004/kjnt.2016.12.2.144 27857924 PMC5110905

[16] Shin T Kim JH Kwak KW Kim SH. Transorbital penetrating intracranial injury by a chopstick. J Korean Neurosurg Soc. (2012) 52:414–6. 10.3340/jkns.2012.52.4.414 23133735 PMC3488655

[17] Yamashita T Mikami T Baba T Minamida Y Sugino T Koyanagi I. Transorbital intracranial penetrating injury from impaling on an earpick. J Neuro Ophthalmol. (2007) 27:48–9. 10.1097/WNO.0b013e3180325ef4 17414874

[18] Umansky F Nathan H. The lateral wall of the cavernous sinus. With special reference to the nerves related to it. J Neurosurgery. (1982)(56): 228–34. 10.3171/jns.1982.56.2.0228 7054432

[19] Al-Otaibi F Baeesa S. Occult orbitocranial penetrating pencil injury in a child. Case Rep Surg. (2012) 2012:716791. 10.1155/2012/716791 23304618 PMC3529444

[20] Parajuli A Bastola B Shrestha G Jyoti Baba Shrestha J. A case of transorbital intracranial injury presenting with subtle neurological deficit. Nepal J Ophthalmol. (2015) 7:186–90. 10.3126/nepjoph.v7i2.14977 27363966

[21] Dalley RW. Intraorbital wood foreign bodies on CT: use of wide bone window settings to distinguish wood from air. Am J Roentgenol. (1995) 164:434–5. 10.2214/ajr.164.2.7839984 7839984

[22] Specht CS Varga JH Jalali MM Edelstein JP. Orbitocranial wooden foreign body diagnosed by magnetic resonance imaging. Dry wood can be isodense with air and orbital fat by computed tomography. Surv Ophthalmol. (1992) 36:341–4. 10.1016/0039-6257(92)90110-F 1566235

[23] Zenter J Hassler W Petersen D. A wooden foreign body penetrating the superior orbital fissure. Neurochirurgia. (1991) 34:188–90. 10.1055/s-2008-1053489 1775211

[24] Sadasivan B Ma SH Dujovny M Ausman JI Zamorano L Dragovic L. Anterior cavernous sinus space. Acta Neurochir. (1991) 108:154–8. 10.1007/BF01418524 2031475

[25] Yasuda A Campero A Martins K Rhoton A Oliveira E Ribas G. Microsurgical anatomy and approaches to the cavernous sinus. Neurosurgery. (2005) 56:4–27. 10.1227/01.NEU.0000144208.42171.02 15799789

[26] Tuccar E Uz A Tekdemir I Ersoy EM Deda H. Anatomical study of the lateral wall of the cavernous sinus, emphasizing dural construction and neural relations. Neurosurg Rev. (2000) 23:45–8. 10.1007/s101430050031 10809487

[27] Kawase T van Loveren H Keller JT Tew JM. Meningeal architecture of the cavernous sinus: clinical and surgical implications. Neurosurgery. (1996) 39:527–34; discussion 534–536. 10.1227/00006123-199609000-00019 8875483

[28] Fukuda H Evins AI Burrell JC Iwasaki K Stieg PE Bernardo A. The meningo-orbital band: microsurgical anatomy and surgical detachment of the membranous structures through a frontotemporal craniotomy with removal of the anterior clinoid process. J Neurol Surg B Skull Base. (2014) 75:125–32. 10.1055/s-0033-1359302 24719799 PMC3969442

[29] Lefebvre DR Chandra RV. Diagnosis and treatment of penetrating orbital cranial foreign body injuries. Digit J Ophthalmol. (2012) 18:9–11. 10.5693/djo.04.2012.06.001 23847449 PMC3687100

[30] Tayebi Meybodi A Lawton MT Yousef S Guo X González Sánchez J Tabani H. Anterior clinoidectomy using an extradural and intradural 2-step hybrid technique. J Neurosurg. (2018)130:238–47. 10.3171/2017.8.JNS171522 29473783

[31] Perneczky A Reisch R Keyhole Approaches in Neurosurgery. Vienna: Springer (1999).

[32] Lohse M Garrido L Driver J Dolan RJ Duchaine BC Furl N. Effective connectivity from early visual cortex to posterior occipitotemporal face areas supports face selectivity and predicts developmental prosopagnosia. J Neurosci. (2016) 36:3821–28. 10.1523/JNEUROSCI.3621-15.2016 27030766 PMC4812138

[33] Pertzov Y Miller TD Gorgoraptis N Caine D Schott JM Butler C. Binding deficits in memory following medial temporal lobe damage in patients with voltage-gated potassium channel complex antibody-associated limbic encephalitis. Brain. (2013) 136:2474–85. 10.1093/brain/awt129 23757763 PMC3722347

[34] Dolenc V. Direct microsurgical repair of intracavernous vascular lesions. J Neurosurgery. (1983) 58:824–31. 10.3171/jns.1983.58.6.0824 6854374

